# The Dentist of Bigler’s Gulch

**Published:** 1882-02

**Authors:** Joachkhim Miller, A.E. Bartlett

**Affiliations:** San Diego, California


### CORRESPONDENCE.

### THE DENTIST OF BIGLER'S GULCH.

A LEGEND OF THE SIERRAS.

BY JOACHKHIM MILLER.

The newly settled little village of BIGLER'S GULCH was startled one morning by the appearance of the following sign: "J. S. Bemis, Dentist. Teeth Extracted Without Pain." When the above straight-forward, unostentatious sign-board first appeared at the top of an outside flight of rickety stairs, leading to an office in the second story of an unpainted, unfinished, unpretending wooden building in the aforesaid village, the miners, and especially the miner's wives and daughters, wondered at the apparent foolhardiness or daring audacity of a stranger, who should unheralded precipitate himself upon an honest, hard-working community, and presume to glean a precarious livelihood in this, to them, professionally effeminate manner.

Doctor Bemis had been in his "office" for nearly five weeks, sustaining himself no one knew how; occasionally seen in the dusk of evening meekly coming with a pitcher of milk through the back gate from a neighbor's house, or going with a small bundle in his hands to his washerwoman, in the suburbs; though he must have lived chiefly on "the sick hope of fees to come," for he was certainly nourished and ministered to in some mysterious and miraculous manner. He was quiet, gentlemanly and unobtrusive in his habits, and mixed but little with the incongruous, uncongenial society that surrounded him; seeming to court seclusion rather than notoriety.

It was a gloomy, dismal, rainy evening, and as the rain was pouring without, he likewise, in doleful mood, was pouring within over the bloody precepts and homilies of "Harris' Dental Surgery." A loud step startled him! It shook his frame "as if a trumpet rang!" Trembling he closed the book. The step was on the stairs—it advanced, with the heavy muffled tread of a son of Anak! It came on with the stride of an avenger! All his misdeeds came up before him as in the mirror of fate. The stairs groaned---the building shook and quivered at his coming? There was a fearful, muffled, scratching sound, like unto the flapping and folding of gigantic wings!---like unto the claw armed wings of the great Vampire, or Apolyon, at the door! A knock that smote his inmost soul and froze his blood with horror! The door seemed to open instinctively of its own volition; when, at this supreme juncture, in stalked an individual of marked appearance and large proportions, with a wet umbrella in his hand. Immediately there came this exclamation from his mouth, as it were from the mouth of an oracle of Delphi:

"Be yeou ther feller that pulls teeths he-ar without payin'? Yas---wal, I reckoned so by ther look er yer pullin' irons reound he-ar. How der yer git 'em eout? Du yer yerk 'em, or flop em? eh? I like floppin' on em best, if yer man is only hefty and 'll hold together. Well, look ter he-ar stranger; I've got er lot uv harf a duzzin or so that I'll let yer yerk or flop or wrassel with, seein' yeou doant seem to be busy like!"

He accordingly seated himself bolt upright upon the floor, straight as a try-square, but finally gathered himself up and adjusted himself in the chair of torture, and the doctor, who had by this time in some measure regained his composure, finally succeeded, by dint of yerkin' and floppin', according to the occasional directions of the patient himself, in wrenching from the bony strata where they were imbedded seven gigantic molars, bicuspids, incisors, and "asteroids," that would not have seemed unseemly in the jaws of a mastodon, or fabled animal of the ante-carboniferous or silurian epochs, and which would have taxed the power of one of Parish's patent stump-pulling machines itself. After cooly gathering up the seven bloody trophies of the occasion in his hand and then putting them into his pocket, he remarked substantially as follows:

"I spose a philanthrofistical kind of a feller like you don't kere to keep these ere grinders; and I'm sure I'm bleeged to yer, young chap, for yerkin' on 'em out fer nothin', fer the've pestered my old woman nights orful, she says, by my grinding' and chawin' and groanin', and I shudn't er com'd up ef I hadn't er seed yer sine, a month er tew ago, 'Teeth extracted without payin',' and I tho't then ef ever I had occasion I'd patronize yer; fer I liked yer style of doin' bisniss and hoped you'd hold on here till you'd bilt up a bizniss, and here's hopin' you'll succeed. You're a clever feller, any how, and purty powerful at a yerk, if I do say it, but I don't see how you make it pay, for our folks say you don't have much customers; but I wish you well, and hope you'll stay among us an' be encouraged, and we'll dew what we can fer yer."

With these friendly remarks he resumed his wet umbrella and departed down the stairs, in the same measured, methodical, and solemn manner in which he came. The next day the office of Doctor Bemis was deserted, and no man to this day knoweth the place of his present sojourn; yet his sign still hangs out and creaks upon its rusty hinges, in the view of a perverse and unappreciative and gainsaying generation.

A. E. Bartlett.

San Diego, California, Oct. 10, 1881.

